# Impact of aging on the frequency, phenotype, and function of CD4+ T cells in the human female reproductive tract

**DOI:** 10.3389/fimmu.2024.1465124

**Published:** 2024-09-12

**Authors:** Zheng Shen, Landon G. vom Steeg, Mickey V. Patel, Marta Rodriguez-Garcia, Charles R. Wira

**Affiliations:** ^1^ Department of Microbiology and Immunology, Geisel School of Medicine at Dartmouth, Lebanon, NH, United States; ^2^ Department of Biochemistry, Microbiology and Immunology, Wayne State University School of Medicine, Detroit, MI, United States; ^3^ C. S. Mott Center for Human Growth and Development, Department of Obstetrics and Gynecology, Wayne State University School of Medicine, Detroit, MI, United States

**Keywords:** aging, menopause, human female reproductive tract, CD4+ T cells, tissue resident memory phenotype T cells, cytokine production

## Abstract

Since CD4+ T cells are essential for regulating adaptive immune responses and for long lasting mucosal protection, changes in CD4+ T cell numbers and function are likely to affect protective immunity. What remains unclear is whether CD4+ T cell composition and function in the female reproductive tract (FRT) changes as women age. Here we investigated the changes in the composition and function of CD4+ T cells in the endometrium (EM), endocervix (CX), and ectocervix (ECX) with aging. We observed a significant decrease in both the total number and percentage of CD4+ T cells in the EM with increasing age, particularly in the years following menopause. CD4+ T cells within the FRT predominantly expressed CD69. The proportion of CD69+CD4+ T cells increased significantly with increasing age in the EM, CX and ECX. The composition of T helper cell subsets within the EM CD4+ T cell population also showed age-related changes. Specifically, there was a significant increase in the proportion of Th1 cells and a significant decrease in Th17 and Treg cells with increasing age. Furthermore, the production of IFNγ by CD4+ T cells in the EM, CX, and ECX significantly decreased with increasing age upon activation. Our findings highlight the complex changes occurring in CD4+ T cell frequency, phenotype, and function within the FRT as women age. Understanding these age-related immune changes in the FRT is crucial for enhancing our knowledge of reproductive health and immune responses in women.

## Introduction

The elderly population, defined as individuals aged 60 years and older, is experiencing a rapid and significant increase, projected to reach 1.4 billion by 2030. Among this demographic, women constitute approximately two-thirds of the population (1). Older women encounter distinct health challenges, including increased risk of urinary tract infections (UTIs), sexually transmitted infections (STIs), and gynecological cancers increasing as well (2, 3).

As the first line of defense, the mucosal immune system of the human female reproductive tract (FRT) protects against both gynecological cancers and infections by a spectrum of sexually transmitted pathogens, including Human Immunodeficiency Virus (HIV) (4, 5). Both the innate and adaptive immune protection in the FRT is known to be precisely controlled by hormonal changes during the menstrual cycle and pregnancy (6). In contrast, little is known about the alterations in mucosal immune protection in the FRT as women age following menopause.

Since the average age at menopause is 52 years in the United States (7), and the average life expectancy of women is 81 years (8), this results in women having a uniquely long survival potential in a post-menopausal environment characterized by low concentrations of sex hormones (9). When reproductive function ends, two overlapping processes contribute to changes in immune protection in aging women: the low sex hormone environment following the onset of menopause and age-related changes to the immune system (10). A major gap in our knowledge is understanding the extent to which innate and adaptive immunity throughout the FRT are altered following menopause and with increasing age.

Central to adaptive immune protection in the FRT are the T cells which are a major constituent of leukocytes (30–60%) (11–13). Within this population, CD4+ T cells represent 35-50% of total CD3+ cells in the FRT. Following menopause, CD4+ T cell presence is significantly reduced in the endometrium (EM) but not the endocervix (CX) and ectocervix (ECX) (12). In addition, the expression of CCR5 is increased on FRT CD4+ T cells following menopause (12, 14). CCR5 is a chemokine receptor with important roles in reproductive function and the primary coreceptor used by HIV to infect genital tissues (15). Changes in FRT CD4+ T cells composition and function with aging, and the potential consequences for immune protection remain unknown.

Relevant to mucosal sites is the presence of tissue resident memory T cells (TRMs), which remain in tissues without recirculating, thereby providing first line local defense against reinfection and reactivation (16). We and others have demonstrated that a high proportion of human FRT T cells express the tissue residency markers CD69 and CD103 (13, 17–20). TRMs remain constant throughout the life span in multiple organs and mucosal surfaces (21). However, in the FRT, we previously found that CD103+ T cells presence significantly change with menopause and aging in a site-specific manner (13). For example, EM CD103+ T cells increase after menopause and remain constant with post-menopausal aging. In contrast, in the CX, CD103+ T cells progressively declined after menopause as women age. Age-related changes in the FRT were specific to CD103+CD8+ T cells, with no modifications on CD103+CD4+ T cell expression, which represents less than 10% of the CD103+ T cell population. The extent to which aging affects CD69 expression on CD4+ T cells is unknown.

Here, using hysterectomy surgery samples from women ranging from 28 to 75 years of age, we investigated the changes in composition and function that occur in FRT CD4+ T cells with aging. We found that aging leads to profound changes in FRT CD4+ T cell numbers, frequency, phenotype, and the production of cytokines. Understanding the underlying factors and mechanisms involved in regulating cell-mediated protection by CD4+ T cells from FRT will contribute to the foundation of information essential for developing therapeutic tools to protect women against gynecological cancers and sexually transmitted infections as they age in the years following menopause.

## Materials and methods

### Study subjects

Human endometrium (EM), endocervix (CX) and ectocervix (ECX) tissues were obtained following hysterectomy surgery at Dartmouth-Hitchcock Medical Center (Lebanon, NH). Studies were performed with approval from the Dartmouth College Institutional Review Board (IRB), Committee for the Protection of Human Subjects (CPHS), Dartmouth Health (DH), and with written informed consent obtained from the patients before surgery. All investigations were conducted according to the principles expressed in the Declaration of Helsinki. Indications for surgery were benign conditions such as fibroids and prolapse (age from 28-75). Selected tissues were distal from the sites of pathology and were without pathological lesions as determined by a pathologist from DH. Tissues were only included from women not on oral contraceptives or post-menopausal hormone therapy prior to hysterectomy. Menopausal status was determined by a pathologist based on the histological evaluation of sections of the EM (endometrial dating). Post-menopausal status was defined as an atrophic EM. Information regarding genital infections was not available.

### Tissue processing

Tissues were transferred to the laboratory immediately after surgery and processed as previously described (13, 18, 22, 23). The average tissue weight obtained was 4.0 ± 2.9 grams. Tissues were rinsed with HBSS (Hank’s balanced salt solution) supplemented with phenol red, 100 U/ml penicillin, 100 µg/ml streptomycin, and 0.35 mg/ml NaCO_3_ (all Thermo Fisher Scientific, Waltham, MA). Tissues were then minced under sterile conditions into 1-2 mm fragments and digested using an enzyme mixture containing 0.05% collagenase type IV (Sigma-Aldrich, St. Louis, MO) and 0.01% Deoxyribonuclease I (Worthington Biochemical, Lakewood, NJ) in HBSS for 1h at 37°C in a humidified 5% CO2 incubator. Type IV collagenase was selected based on studies to ensure non-cleavage of surface markers (12, 24). After digestion, cells were dispersed through a 250 µm nylon mesh filter (Small Parts, Miami Lakes, FL) followed by sequential filtration of the flow-through through 40 and 20 µm nylon mesh filters (Small Parts). Epithelial cell sheets were retained on the filters, while stromal cells passed through. Stromal cells were resuspended in culture medium X-VIVO 15 with Phenol Red Media (Lonza, Walkersville, MD) supplemented with 10% human serum (BioIVT, Westbury, NY) and cultured overnight at 37°C in a humidified 5% CO2 incubator. After overnight recovery, cells were washed, erythrocytes were lysed, and dead cells were removed using the Dead Cell Removal Kit (Miltenyi Biotec, Auburn, CA) as previously described (23, 25). This process achieved over 90% cell viability, as determined by flow cytometry with the Live/Dead Fixable Yellow Dead Cell Stain Kit (Thermo Fisher Scientific), as shown in **Supplementary Figure 1**. The resulting mixed cell suspension, consisting of immune cells and stromal fibroblasts, was used to continue CD4+ T cell purification or perform flow cytometry staining for phenotyping and functional analysis.

### Isolation of FRT CD4+ T cells

Following removal of dead cells, CD4+ T cells were isolated using negative magnetic bead selection with the CD4+ T cell isolation kit (Miltenyi Biotec) following instructions with minor modifications. This negative selection protocol delivers untouched CD3+CD4+ T cells. Additionally, anti-fibroblast microbeads (Miltenyi Biotec) were added in combination with the microbeads supplied with the kit to ensure depletion of stromal fibroblasts present in the mixed cell suspension as previously described (12). After two rounds of negative selection, purity of the CD4+ T cell population was higher than 90%. Following isolation, viable purified CD4+ T cells were counted using trypan blue (Thermo Fisher Scientific) by hemocytometer on a light microscope with 10X objective and calculated the number of CD4+ T cells per gram of tissue in each patient.

### Flow cytometry

Following dead cell removal, mixed cell suspensions were washed twice with PBS containing 10% of human serum (BioIVT). For phenotyping analysis, cells were stained for surface antibodies (**Supplementary Table 1**) for 30 min at 4°C in the dark. For functional analysis, cells were stained with surface antibodies for 30 min at 4°C in the dark, monoclonal antibodies for intracellular staining (**Supplementary Table 2**) were added for 30 min at 4°C in the dark after fixation and permeabilization of the cells using Cytofix/Cytoperm Reagent set (BD Biosciences, Franklin Lakes, NJ) according to instructions to detect the production of IFNγ, IL-4, IL-17A, IL-22 or intracellular expression of CTLA-4. Analysis was performed on Gallios flow cytometers (Beckman Coulter, Indianapolis, IN) using Kaluza software, and data analyzed with FlowJo software (Tree Star, Inc. Ashland, OR). Expression of surface and intracellular markers was measured by the percentage of positive cells. Florescence minus one (FMO) was used for gate setting.

### Functional analysis

Mixed cell suspensions were stimulated with phorbol 12- myristate 13-acetate (PMA) (100 ng/ml, Abcam), ionomycin (2 µM, Calbiochem). Medium alone was added as the unstimulated negative control. All incubations were performed in the culture medium in the presence of brefeldin A and monensin (eBioscience™ protein transport inhibitor cocktail, Thermo Fisher Scientific) according to instructions for 6h at 37°C in a humidified 5% CO2 incubator. Functionality was determined by measuring the intracellular production of IFN-γ, IL-4, IL-17A, and IL-22 by flow cytometry as described above.

### Statistics

Data analysis was performed using the GraphPad Prism 9 (GraphPad Software, San Diego, CA). A two-sided P value <0.05 was considered statistically significant. Comparison of two groups was performed with the nonparametric Mann–Whitney U-test. Comparison of three or more groups was performed applying the non-parametric Kruskal-Wallis test or the paired Friedman test followed by Dunns post-test. Correlation analyses were performed applying nonparametric Spearman test.

## Results

### EM CD4+ T cell numbers per gram of tissue decline significantly with increasing age following menopause

**Figure 1:**
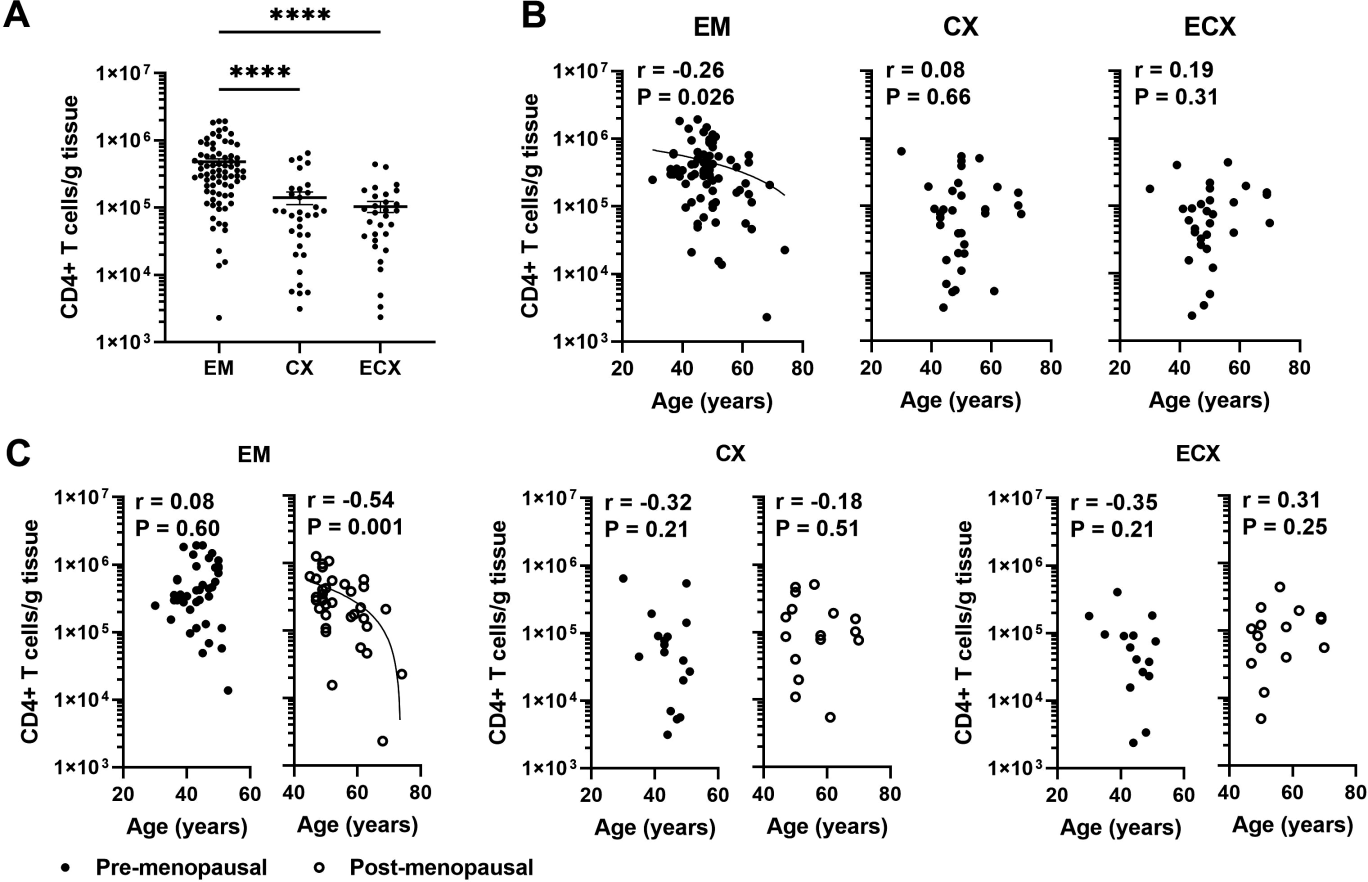
EM CD4+ T cell numbers per gram of tissue decline significantly with increasing age following menopause. **(A)** Number of CD4+ T cells recovered per gram of tissue from endometrium (EM; n=75), endocervix (CX; n=33) and ectocervix (ECX; n=30) after magnetic bead isolation. **(B)** Correlation between age and number of CD4+ T cells recovered per gram of tissue in the EM (n=75), CX (n=33) and ECX (n=30) from the entire study population. **(C)** Correlation between age and number of CD4+ T cells recovered per gram of tissue in the EM, CX and ECX from pre- (black circle; EM: n=40, CX: n=17, ECX: n=15) or post-menopausal (white circle; EM: n=35, CX: n=16, ECX: n=15) women. Each dot represents a different patient. Mean ± SEM are shown. ****P<0.0001; Kruskal-Wallis test followed by Dunns post-test **(A)**, Spearman test **(B, C)**.

Previous studies from our laboratory have demonstrated that in pre-menopausal women the total leucocyte population in the EM remains either unchanged or increases slightly during the menstrual cycle, followed by a decline after menopause (11). We have also shown that dendritic cell and CD8+ T cell numbers in the EM decline with increasing age in a combined population of pre- and post-menopausal women (13, 20). To understand the dynamics of cell-mediated immunity more fully in the years following menopause, we measured the number of CD4+ T cells per gram of FRT tissue in patients after negative magnetic bead selection. As seen in **Figure 1A**, across all patients, we recovered an average of 4.79x10^5^, 1.41x10^5^, and 1.04x10^5^ number of CD4+ T cells per gram of tissue from the EM, CX, and ECX respectively. The density of CD4+ T cells per gram of tissue was significantly higher in the EM compared to the CX and ECX. To investigate if menstrual status and patient age affects CD4+ T cells number in FRT tissues, we stratified tissues based on pre- or post-menopausal status, as established by EM tissue histology. We found that EM CD4+ T cells numbers from post-menopausal women were significantly lower than those in pre-menopausal women (**Supplementary Figure 2A**). In pre-menopausal EM tissue, we recovered significantly more cells (5.88x10^5^ cells/g) than in post-menopausal tissue (3.54x10^5^ cells/g). In contrast to the EM, there were no significant effects of menopausal status observed on the number of CD4+ T cells in the CX or ECX (**Supplementary Figure 2A**). We then examined the correlation between age and CD4+ T cell numbers across the entire study population, specifically within both the pre- and post-menopausal populations. As seen in **Figure 1B**, we observed a significant decrease in CD4+ T cell density in the EM with increasing age across the entire population, whereas no significant changes were found in the CX and ECX. Within the post-menopausal population, EM CD4+ T cell density showed a significant decrease with increasing age (**Figure 1C**). In contrast, there was no significant age-related change in the number of EM CD4+ T cells within the pre-menopausal population. Furthermore, the number of CD4+ T cells per gram of tissues in the CX and ECX did not show significant changes with age in either pre- or post-menopausal populations (**Figure 1C**).

### Aging beyond menopause decreases the frequency of EM CD4+ T cells in the CD3 population

**Figure 2:**
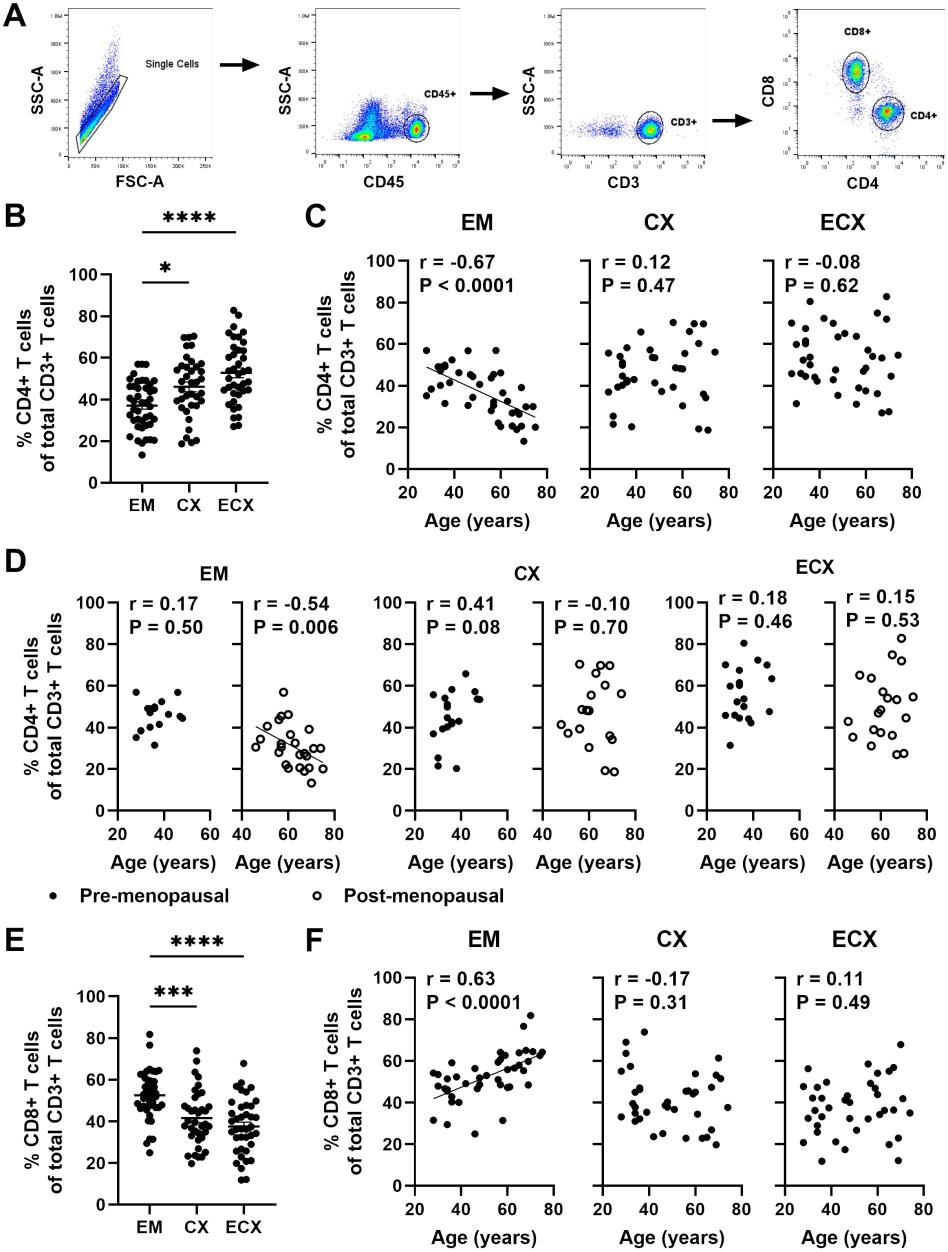
Aging beyond menopause decreases the frequency of EM CD4+ T cells. **(A)** Representative plot of the gating strategy to select CD4+ T cells and CD8+ T cells within the CD3+ T cell population. **(B)** Percentage of CD4+ T cells within CD3+ T cells in endometrium (EM; n=43), endocervix (CX; n=37) and ectocervix (ECX; n=39). **(C)** Correlation between age and percentage of CD4+ T cells within CD3+ T cells in the EM (n=43), CX (n=37) and ECX (n=39) from the entire study population. **(D)** Correlation between age and percentage of CD4+ T cells within CD3+ T cells in the EM, CX and ECX from pre- (black circle; EM: n=18, CX: n=19, ECX: n=19) and post-menopausal (white circle; EM: n=25, CX: n=18, ECX: n=20) women. **(E)** Percentage of CD8+ T cells within CD3+ T cells in in the EM (n=43), CX (n=37) and ECX (n=39). **(F)** Correlation between age and percentage of CD8+ T cells within CD3+ T cells in the EM (n=43), CX (n=37) and ECX (n=39). Each dot represents a different patient. Mean ± SEM are shown. *P<0.05, ***P<0.001, ****P<0.0001; Kruskal-Wallis test followed by Dunns post-test **(B, E)** Spearman test **(C, D, F)**.

Previous studies from our laboratory have demonstrated that post-menopausal women had a reduced percentage of CD4+ T cells in the CD3 population compared to pre-menopausal women specifically in the EM (12). Since CD4+ T cells play a crucial role in orchestrating adaptive immune responses, changes in their frequency are likely to affect protective immunity in the FRT. However, the extent to which aging affects the frequency of FRT CD4+ T cells is unknown. FRT tissues were digested as described in methods to obtain mixed cell suspension. We characterized the frequency of CD4+ T cells within the CD3+ T cell population in the EM, CX and ECX using flow cytometry; a representative example of the gating strategy is shown in **Figure 2A**. Consistent with our previous findings (12), we found that CD4+ T cells accounted for approximately 37% of the total CD3+ cells present in EM, while in CX and ECX, they constituted 46% and 52%, respectively, of the total CD3+ cells (**Figure 2B**). The frequency of CD4+ T cells within the CD3+ cell population was significantly lower in the EM compared to the CX and ECX (**Figure 2B**). In addition, we found that the percentage of CD4+ T cells was significantly higher in pre-menopausal women compared to post-menopausal women in the EM (46% vs 31%), but not in the CX (45% vs 47%) and ECX (55% vs 50%) (**Supplementary Figure 2B**). To investigate if the patient age impacted CD4+ T cell frequency in FRT tissues, we examined the percentage of CD4+ T cells as a function of age across the entire study population, as well as within pre- and post-menopausal populations. As seen in **Figure 2C**, the percentage of CD4+ T cells among CD3+ T cells significantly decreased with increasing age in the EM of the entire population, but not in the CX and ECX. There was a significant decrease in the percentage of CD4+ T cells with increasing age following menopause in the EM, whereas no such correlation was observed prior menopause (**Figure 2D**). In contrast, no age-related effects were observed on the percentage of CD4+ T cells in CX and ECX, regardless of menopausal status (**Figure 2D**).

We have reported a decline in the number of CD8+ T cells in the EM as women age (20). It would be valuable to investigate how the frequency of CD8+ T cells within the CD3+ T cell population changes with age. As shown in **Figure 2E**, the frequency of CD8+ T cells was significantly higher in the EM compared to the CX and ECX, with approximately 52%, 42%, and 38% in EM, CX, and ECX, respectively. Additionally, the percentage of CD8+ T cells among CD3+ T cells significantly increased with age in the EM across the entire population, whereas no such change was observed in the CX and ECX (**Figure 2F**). This suggests that the number of CD8+ T cells in the EM does not decrease as rapidly as the CD4+ T cells with aging.

### Aging enhances the frequency of tissue resident CD69+CD4+ T cells from FRT tissues

**Figure 3:**
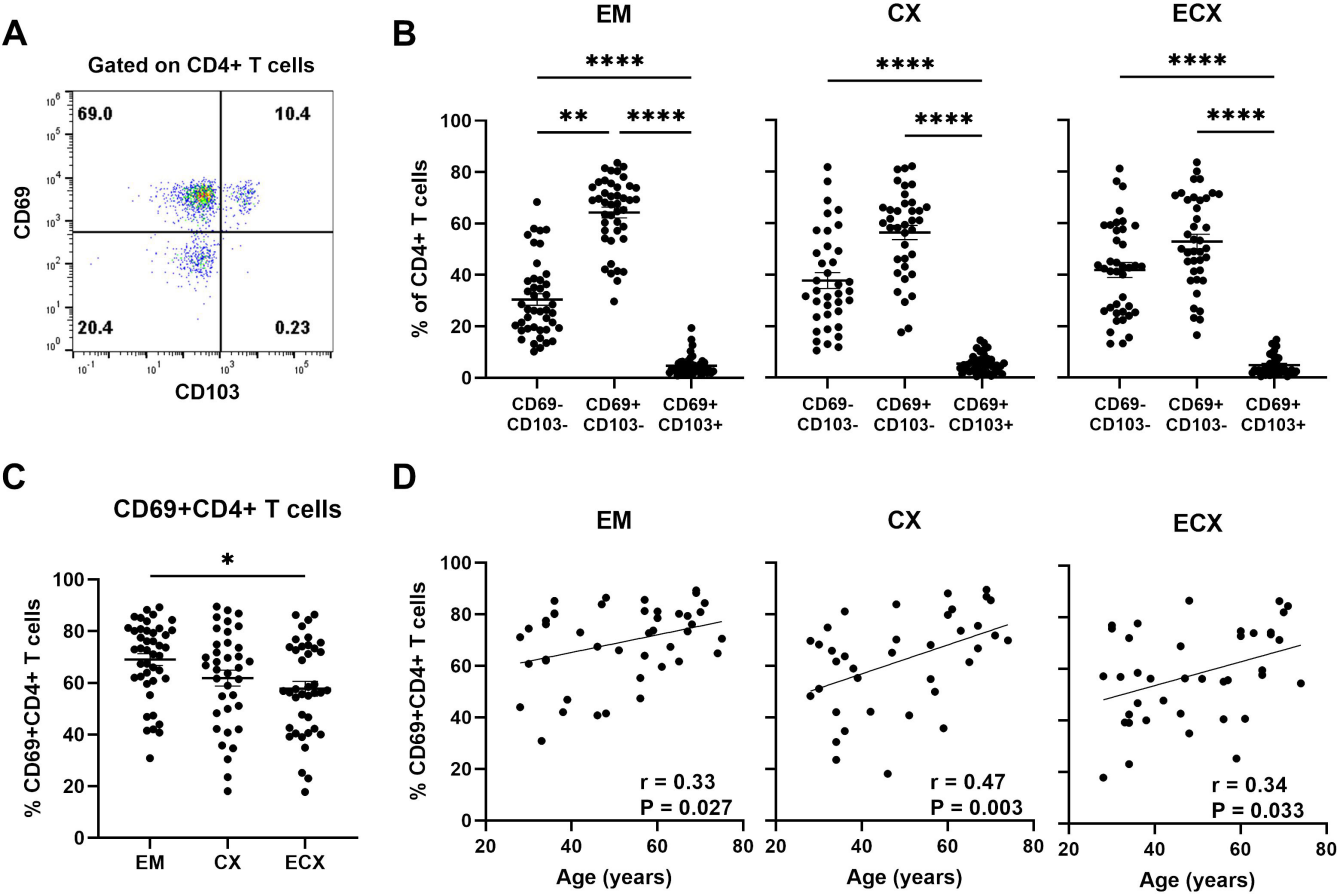
Aging enhances the frequency of tissue resident CD69+CD4+ T cells from FRT tissues. **(A)** Representative plot of the expression of CD69 and CD103 on CD4+ T cells from FRT. **(B)** Percentage of CD69+ and CD69- with or without CD103 cells found on CD4+ T cells in endometrium (EM; n=43), endocervix (CX; n=37) and ectocervix (ECX; n=39). **(C)** Percentage of CD69+CD4+ T cells (combined CD69+CD103+ and CD69+CD103- cells) from EM, CX and ECX. **(D)** Correlation between age and percentage of CD69+CD4+ T cells from EM, CX and ECX. Each dot represents a different patient. Mean ± SEM are shown. *P<0.05, **P<0.01, ****P<0.0001; Friedman test followed by Dunns post-test **(B)**, Kruskal-Wallis test followed by Dunns post-test **(C)**, Spearman test **(D)**.

CD69 and CD103 are commonly used as biomarkers to identify human resident memory T cells, each thought to have a unique contribution towards establishing residency and displaying different expression patterns in human non-lymphoid tissues (26, 27). We have demonstrated previously that in the FRT, CD69 is broadly expressed on CD4+ and CD8+ T cells, while CD103 is preferential to CD8+ T cells and co-expressed with CD69, and the proportion of CD103+CD4+ T cells is very low and does not change with age (10, 13). Since the extent to which aging affects CD69 expression on CD4+ T cells is unknown, expression of CD69 and CD103 on CD4+ T cells was analyzed using flow cytometry in different regions of the FRT (EM, CX and ECX); a representative example is shown in **Figure 3A**. Consistent with our previous findings (13), we found that the majority of CD4+ T cells in the EM (64.3%), CX (56.4%), and ECX (52.9%) were CD69 single positive with less than 10% expressing CD103 (**Figure 3B**). Thus, tissue residency in the FRT was defined solely by the expression of CD69, which is consistent with the literature (27). The percentage of CD69+ (both CD103+ and CD103-) CD4+ T cells were abundant in the FRT and varied among samples and across FRT tissue sites. The mean expression frequency of CD69 on CD4+ T cells from EM, CX and ECX was 69.1%, 61.9% and 57.8% respectively, with a significantly higher proportion observed in EM relative to ECX (**Figure 3C**). We then evaluated changes in the percentage of CD69+CD4+ T cells as a function of age (28-75 years) and found that the percentage of CD69+CD4+ T cells increased significantly with increasing age in the EM, CX, and ECX (**Figure 3D**).

### Aging decreases the frequency of regulatory T cells from EM tissues

**Figure 4:**
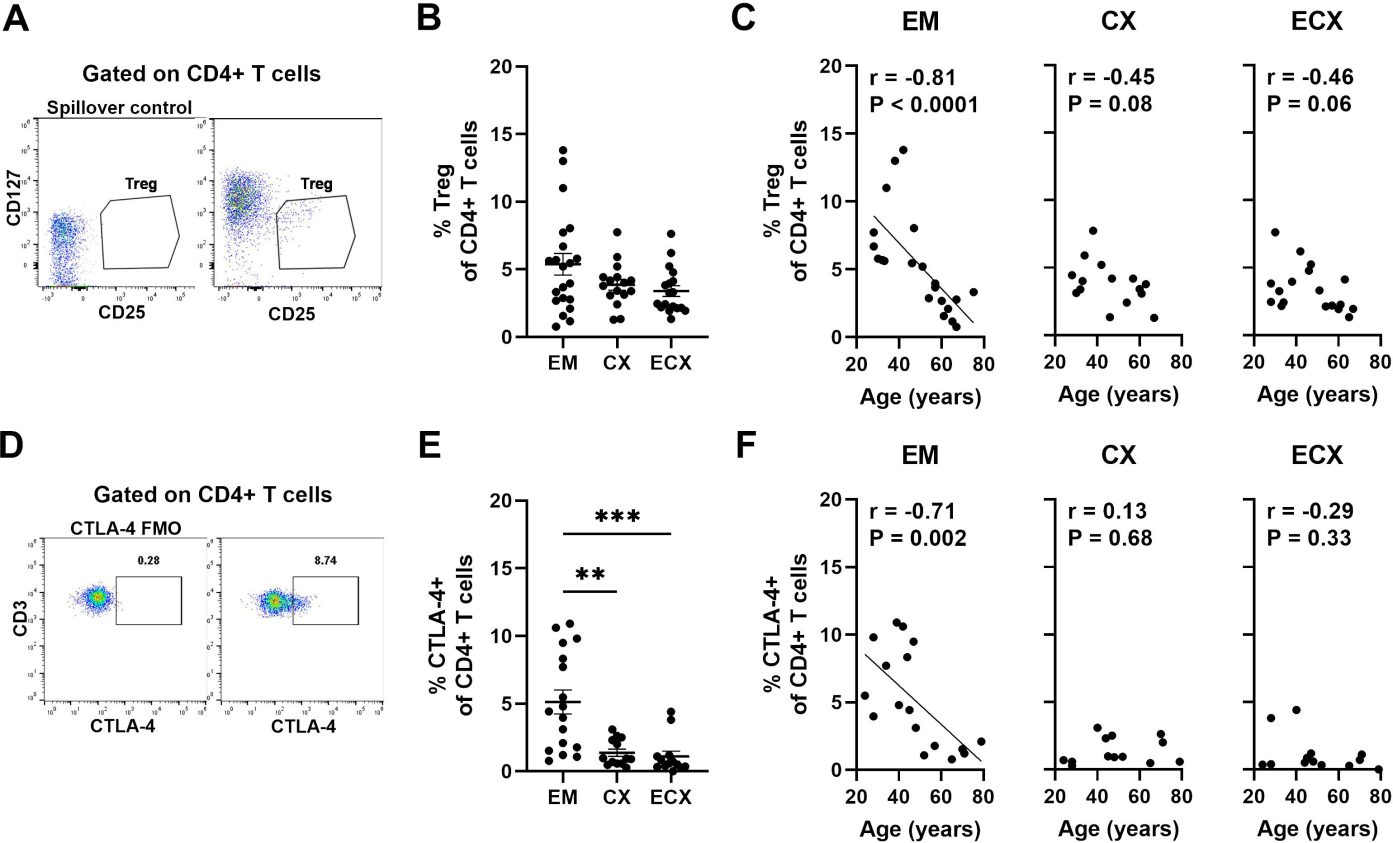
Aging decreases the frequencies of regulatory T (Treg) cells in the FRT. **(A)** Representative gating for CD25 and CD127 expression on CD4+ T cells to phenotypically characterize Treg cells from FRT. The gating was established by a “spillover control” sample that lacked CD25 and CD127. Treg cells were defined as CD25+CD127^low^ cells. **(B)** Percentage of Treg cells found on CD4+ T cells in each EM, CX and ECX tissue and **(C)** Correlation between age and percentage of Treg cells from EM, CX and ECX. EM (n=21), CX (n=16), ECX (n=18). **(D)** Representative plot showing intracellular staining for CTLA-4 on CD4+ T cells from FRT. Negative control was established using fluorescence minus one (FMO). **(E)** Percentage of CTLA-4 expression found on CD4+ T cells in each EM, CX and ECX tissue and **(F)** Correlation between age and percentage of CTLA-4 expression cells from EM, CX and ECX. EM (n=17), CX (n=13), ECX (n=13). Each dot represents a different patient. Mean ± SEM are shown. **P<0.01, ***P<0.001; Kruskal-Wallis test followed by Dunns post-test **(B, E)**, Spearman test **(C, F)**.

Human FRT regulatory T (Treg) cells are essential for maintaining immune tolerance and homeostasis as well as controlling local inflammation to prevent immunopathology (28). The age-related changes of Treg cells are of particular interest (29, 30). Researchers have shown that the percentage and function of Treg change in aged people with higher frequency of Tregs in the skin of old people compared to their younger counterparts (31). However, the extent to which aging affects the frequency of Treg in the FRT is unknown. Tregs were defined as live, single CD3+CD4+ T cells expressing CD25 but low CD127 by flow cytometry (32); representative examples are shown in **Figure 4A**. The percentage of Treg within CD4+ T cells varied across individual samples from EM, CX and ECX, with an average percentage of approximately 3-5%. There were no significant differences in Treg percentages between different sites (**Figure 4B**). We then determined the percentage of Treg cells as a function of age across the entire study populations and found that the percentage of Treg cells in the EM decreased significantly with increasing age. However, there was a trend towards a decrease in the percentage of Treg cells in the CX (P=0.08) and ECX (P=0.06) as women aged, although this trend did not reach statistical significance (**Figure 4C**).

An essential marker for Treg cells is cytotoxic T-lymphocyte-associated protein 4 (CTLA-4) which functions as an immune checkpoint regulator to modulate immune responses (33). It is a predominantly intracellular protein that is constitutively expressed and plays a key role in Treg-mediated suppression (34). Intracellular CTLA-4 expression was analyzed on CD4+ T cells using flow cytometry; representative examples are shown in **Figure 4D**. The percentage of CTLA-4+CD4+ T cells varied significantly across sites in the FRT, with a higher proportion observed in the EM compared to the CX and ECX, averaging 5.1%, 1.4%, and 1.1%, respectively (**Figure 4E**). When we investigated if age of the patients affects the percentage of CTLA-4+CD4+ T cells in FRT tissues, we found that the percentage of CTLA-4+CD4+ T cells in the EM decreased significantly with increasing age. In contrast, no significant changes with age were observed in the CX and ECX (**Figure 4F**).

### Aging selectively impacts the frequency of CD4+ T helper cell subsets in the FRT

**Figure 5:**
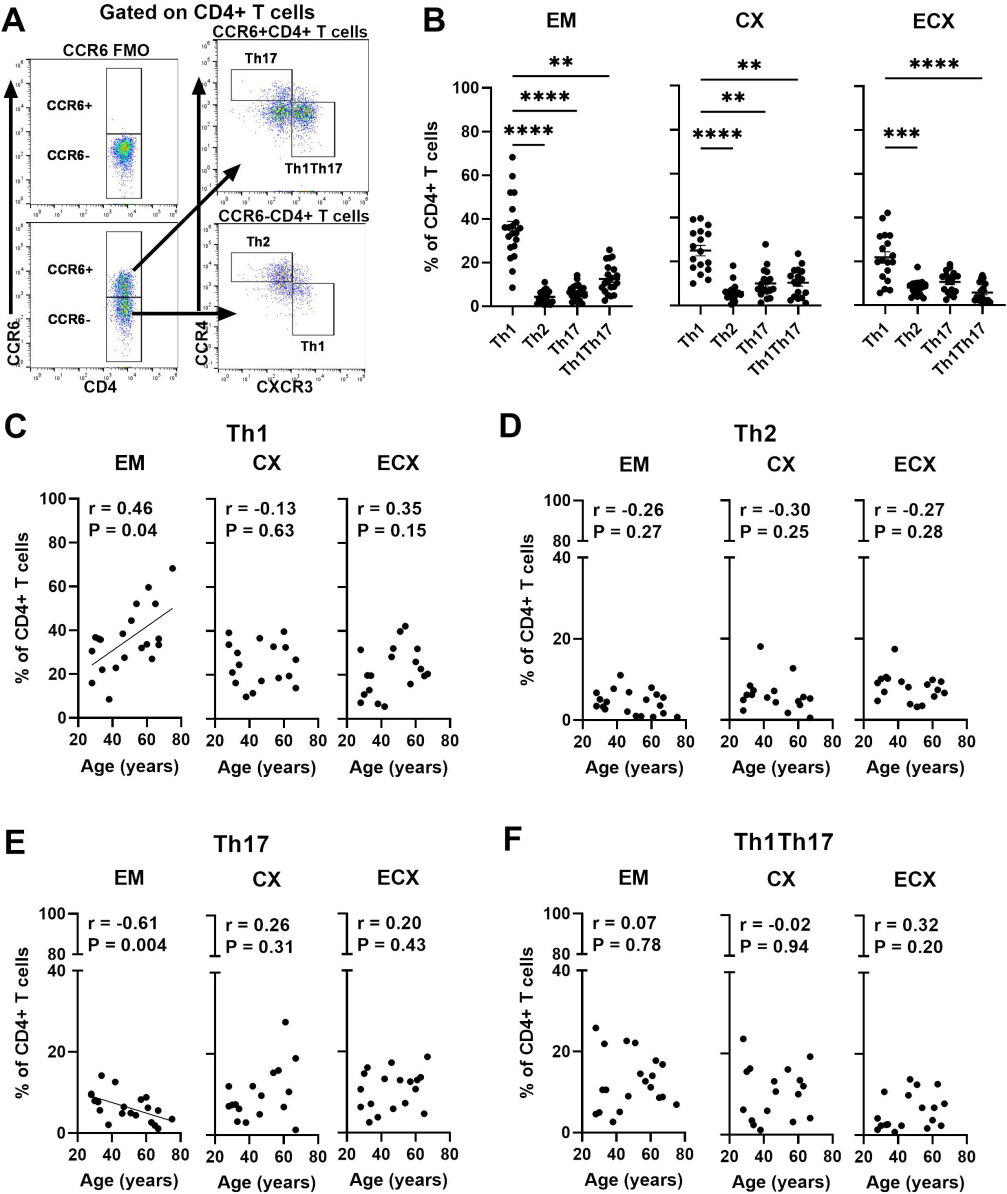
Aging selectively impacts the frequency of CD4+ T helper cell subsets in the FRT. **(A)** Representative gating for CCR6, CXCR3 and CCR4 on CD4+ T cells to phenotypically characterize T helper cell subsets from FRT. Negative control was established using fluorescence minus one (FMO). Populations were defined as Th1 (CCR6-CCR4-CXCR3+), Th2 (CCR6-CCR4+CXCR3-), Th17 (CCR6+CCR4+CXCR3-) and Th1Th17 (CCR6+CCR4-CXCR3+). **(B)** Percentage of Th1, Th2, Th17 and Th1Th17 cell subsets found on CD4+ T cells in each EM, CX and ECX tissue. Correlation between age and percentage of Th1 **(C)**, Th2 **(D)**, Th17 **(E)** and Th1Th17 **(F)** cell subsets from EM, CX and ECX. Each dot represents a different patient. Mean ± SEM are shown. EM (n=20), CX (n=17), ECX (n=18). **P<0.01, ***P<0.001; ****P<0.0001; Friedman test followed by Dunns post-test **(B)**, Spearman test **(C–F)**.

CD4+ T cells are key regulators of the adaptive immune system and can be divided into T helper (Th) cell subsets, including Th1, Th2, Th17 and Th1-like Th17 (Th1Th17) during an immune response based on the expression of different chemokine receptors on CD4+ T cells (35, 36). Since CD4+ T helper cell subsets are fundamental to orchestrating adaptive immune, the frequencies of Th cell subsets were evaluated using surface biomarker staining and multi-color flow cytometry; a representative example is shown in **Figure 5A**. Th cell populations were defined as Th1 (CCR6-CCR4-CXCR3+), Th2 (CCR6-CCR4+CXCR3-), Th17 (CCR6+CCR4+CXCR3-) and Th1Th17 (CCR6+CCR4-CXCR3+), based on their chemokine receptor expression patterns (35). As seen in **Figure 5B**, we found that Th1 cells were the most abundant subset, representing approximately 35.6%, 24.5%, and 21.9% of total CD4+ cells in the EM, CX, and ECX. Th2, Th17, and Th1Th17 cells made up smaller proportions, ranging from 4-8%, 6-11% and 7-13%, respectively in FRT. We next determined the relative frequencies of Th cell subsets as a function of age and found that the frequency of Th1 cells in the EM increased significantly with increasing age, while the frequency of Th17 cells decreased (**Figures 5C, E**). In contrast, there were no significant age-related effects on the frequencies of Th1 and Th17 cells within the CD4+ T cell population from the CX or ECX (**Figures 5C, E**). Additionally, no significant age-related changes were observed in the frequencies of Th2 and Th1Th17 cells throughout the FRT (**Figures 5D, F**).

### Aging selectively decreases the production of IFNγ, IL-17A and IL-22 by CD4+ T cells in the FRT

**Figure 6:**
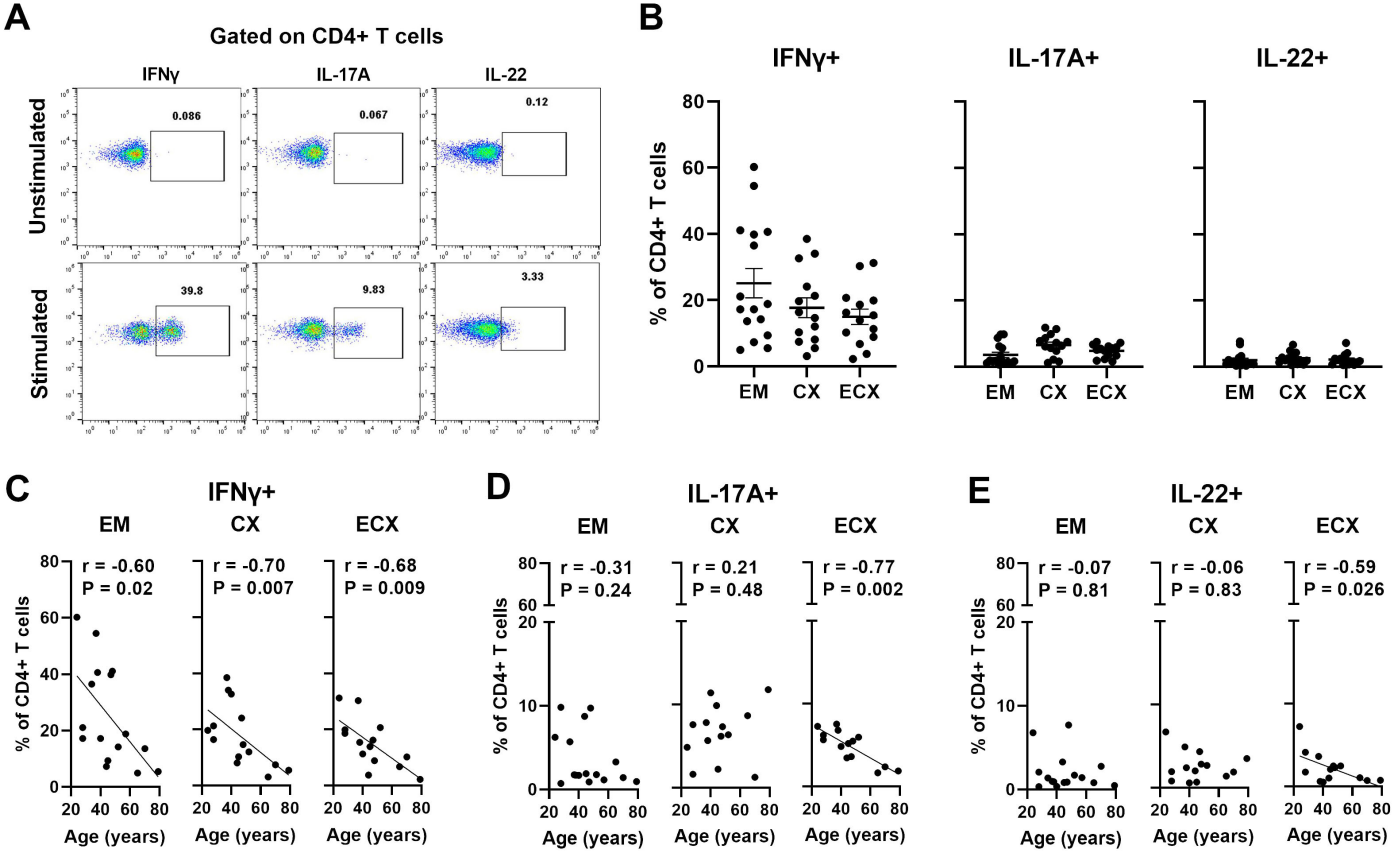
Aging selectively decreases the production of IFNγ, IL-17A, IL-22 by CD4+ T cells in the FRT. **(A)** Representative plot showing intracellular staining for IFNγ, IL-17A, and IL-22 on CD4+ T cells from FRT under 6h unstimulated or PMA+ionomycin stimulated conditions. Medium alone was used as the unstimulated negative control. **(B)** Percentage of IFNγ, IL-17A, and IL-22 expressions found on CD4+ T cells in each EM, CX and ECX tissue. Correlation between age and percentage of IFNγ **(C)**, IL-17A **(D)**, and IL-22 **(F)** expression on CD4+ T cells from EM, CX and ECX. Each dot represents a different patient. Mean ± SEM are shown. EM (n=16), CX (n=14), ECX (n=14). Kruskal-Wallis test followed by Dunns post-test **(B)**, Spearman test **(C–E)**.

Cytokine production is key to CD4 T cells protective immunity (37). Since the extent to which aging affects the functions of FRT CD4+ T cells is unknown, we examined the production of cytokines (IFNγ, IL-4, IL-17A and IL-22) by CD4+ T cells through intracellular staining using multi-color flow cytometry after stimulation with PMA plus ionomycin for 6 hours; representative examples are shown in **Figure 6A**. Medium alone was used as the unstimulated negative control. We found that CD4+ T cells from EM, CX, and ECX produced IFNγ, IL-17A and IL-22 in response to PMA stimulation. The percentages of CD4+ T cells producing IFNγ, IL-17A and IL-22 varied with site sampled in the FRT, averaging 25.1%, 3.6%, and 2.1%, respectively, in the EM; 17.8%, 6.6%, and 2.6%, respectively, in the CX; and 15.0%, 4.9%, and 2.2%, respectively, in the ECX (**Figure 6B**). The production of IL-4 by CD4+ T cells was minimal to undetectable (data not shown). When we investigated if patient age affects the production of IFNγ, IL-17A and IL-22 by CD4+ T cells in FRT tissues, we found that IFNγ production by CD4+ T cells from EM, CX and ECX decreased significantly with increasing age (**Figure 6C**). Interestingly, the production of IL-17A or IL-22 by ECX CD4+ T cells, but not EM and CX CD4+ T cells, also decreased significantly with increasing age (**Figures 6D, E**). Taken together, aging selectively decreased the production of IFNγ, IL-17A and IL-22 by CD4+ T cells in the FRT after activation. These findings suggest that aging impacts the ability of CD4+ T cells in the FRT to mount certain types of immune responses, which could have implications for reproductive health and susceptibility to infections as women age.

## Discussion

**Figure 7:**
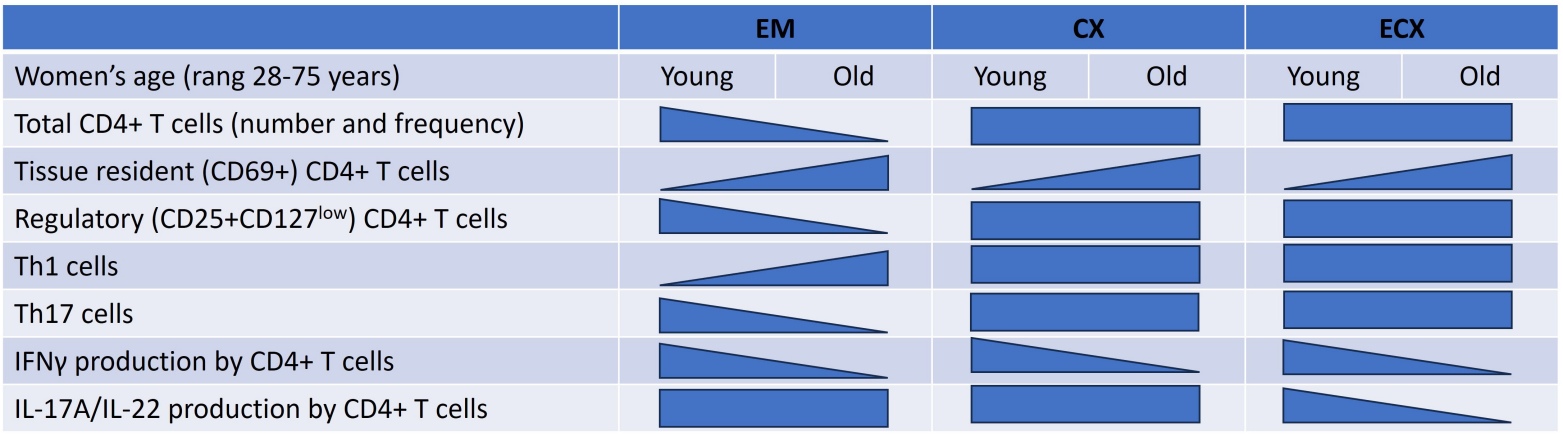
Summary of regulation of CD4+ T cells number, phenotype, and function in the FRT by aging. This diagram indicates FRT CD4+ T cells distribution, phenotype and function that are impacted by women’s age. As indicated by the shape of each triangle, some decline while others increase as women aged. Rectangles indicate no change. Effects are shown for the endometrium (EM), endocervix (CX) and ectocervix (ECX).

Our study demonstrates that changes in composition and function of FRT CD4+ T cells occur with aging. Both the total number of CD4+ T cells per gram of tissue and the percentage of CD4+ T cells in the CD3+ T cell population from the EM significantly decreased with increasing age in both our total population (28-75 years) as well as in the post-menopausal population. In contrast, there were no significant changes observed in the number and percentage of CD4+ T cells in the CX and ECX with age or menopausal status. The majority of CD4+ T cells in FRT expressed CD69, a marker of tissue residency. CD69+CD4+ T cells increased significantly in the EM, CX and ECX with increasing age. Moreover, we found that the composition of T helper cell subsets within the total EM CD4+ T cell population changed with age. Specifically, with increasing age, there was a significant increase in the proportion of Th1 cells and a significant decrease in Th17 and Treg cells. The production of IFNγ by EM, CX, and ECX CD4+ T cells after activation significantly decreased with increasing age (**Figure 7**). Overall, our study provides valuable insights into the complex changes occurring in CD4+ T cell frequency, phenotype, and function within the FRT with aging following menopause. This emphasizes the importance of understanding age-related immune changes in the FRT for reproductive health and immune responses in women.

To understand the dynamics of cell-mediated immunity more fully in the years following menopause, we examined the number of CD4+ T cells per gram of FRT tissue and the percentage of CD4+ T cells in the CD3 population of cells as a function of age and showed, for the first time, that both the number and the percentage of EM CD4+ T cell significantly decreased with increasing age in the years following menopause. This indicates a decline in CD4+ T cells abundance and frequency in the EM with aging, suggesting a potential impact on immune responses in the EM as women age. Our results are consistent with our previous studies where we found the total leucocyte population in the EM declines following menopause (11) and that the frequency of EM CD4+ T cells decreases in post- versus pre-menopausal women (12). We have also previously shown that dendritic cell (DC) and CD8+ T cell numbers in the EM decline with increasing age in a population of pre- and post-menopausal women (13, 20). Our studies extend these observations by demonstrating that CD4+ T cells numbers in EM decline with increasing age following menopause. Whether this decline is due to altered CD4+ T cells recruitment into the EM mucosa or the decrease in ovarian hormones following menopause remains to be determined. In other studies, we found that EM epithelial cells secrete cytokines and chemokines that are essential for regulating the recruitment of immune cells to the FRT (38–40). Furthermore, exposure to epithelial cell secretions can increase the expression of chemotaxis receptors on the surface of immune cells (41). Whether changes in baseline secretion of T cell chemokines such as CCL2, CCL21, RANTES, and SDF-1α by EM cells occur with increasing age remains to be determined but could partly explain why CD4+ T cells decline in the aging EM.

Within the FRT, tissue-resident memory cells (TRMs) play a critical role in providing local protection against recurrent infection and in maintaining tissue homeostasis (17, 27, 42–44). CD69 and CD103 are widely used as biomarkers to identify TRMs (26, 44, 45). Consistent with previous studies (13, 17, 27), we found that the majority of CD4+ T cells in the FRT were single positive for CD69, with fewer than 10% expressing CD103. Therefore, we defined tissue residency based on CD69 expression, a marker of early activation, tissue residency and cell retention (46). Notably, the frequency of CD69+CD4+ T cells increased significantly with increasing age. However, defining TRMs primarily by CD69 expression has limitations. CD69 can be induced by sub-clinical levels of inflammation in the absence of antigen (47). An increase in inflammation with age could in part explain an increase in CD69 expression by CD4+ T cells in the FRT. Additionally, there are likely TRMs that do not express either CD69 or CD103 (48, 49). Furthermore, the observed increase in tissue resident memory CD4+ T cells with age could be influenced by several other factors. For example, with increasing age, women are likely to encounter a wider range of pathogens, leading to increased activation, recruitment and accumulation of memory T cells. Alternatively, in the low sex hormone environment following menopause, the FRT may undergo changes with age, potentially leading to increased retention of activated T cells. Understanding whether inflammation, recruitment, and/or retention contributes to the selective increase in TRMs with age in FRT tissue remains an area for further investigation.

Treg cells are crucial for maintaining immune tolerance and controlling inflammation in the FRT (42, 50, 51). We investigated how aging affects Treg cells frequency throughout the FRT and found that aging selectively decreased the frequency of Treg cells and the constitutive expression of CTLA-4, which helps keep the body’s immune responses in check (34) on resting CD4+ T cells in the EM. Unexpectedly, aging following menopause had no significant effect on the frequency of these cells in the CX and ECX. In pre-menopausal women, the EM immune system must accommodate the reproductive function of the EM and sustain a semi-allogeneic fetus by dampening aspects of adaptive immunity while also maintaining immune protection against pathogens that enter the upper FRT (52, 53). Part of this reproductive function is mediated by Treg cells which exert a strong immunosuppressive effect to maintain an anti-inflammatory environment and protect the fetus from maternal immunological rejection (54). In contrast, the lower tract confers protection against viral and bacterial pathogens, which otherwise might compromise reproductive success (55). Previous studies have demonstrated that endocervical Treg are associated with decreased genital inflammation and low HIV target cell abundance (56). With the transition to menopause and beyond, the reproductive function of the EM ceases, and the balance shifts towards immune protection. The diminished numbers of EM Treg cells and lower expression of CTLA-4 in older women could contribute to an inflammatory mucosal environment. Further studies are needed to identify the mechanisms involved in the transition to an inflammatory EM environment in post-menopausal women.

CD4+ T cells can be divided into T helper (Th) subsets that are involved in humoral and cell-mediated immune responses (36). We investigated the frequency of Th cell subsets based on the expression pattern of chemokine receptors on CD4+ T cells in the FRT and explored how these subsets change with age. The frequency of Th cell subsets in the FRT varied, with Th1 cells being the predominant subset. This is consistent with the finding of others (57). The frequency of EM Th1 cells significantly increased and Th17 cells decreased with increasing age. Interestingly, a consistent proportion of Th1-like Th17 cells was maintained in the FRT as women aged. However, in a previous study (12), we demonstrated an increase in Th17 cells among EM post-menopausal women. This discrepancy may be attributed to our previous focus on CCR6 alone, without analysis of additional chemokine receptors such as CCR4 and CXCR3, which can distinguish Th17 and Th1-like Th17 subsets. Recent studies highlight the plasticity of Th17 cells transforming into Th1-like TH17 cells, a phenomenon recognized in the development of autoimmune and inflammatory conditions (58). These findings suggest that age-related changes in the frequency of Th cell subsets could impact immune function in the FRT, particularly in the EM. Further exploration of these subsets and their functional implications may provide insights into reproductive health and age-related immune dynamics.

An unexpected finding of this study was that CD4+ T cells from older women displayed a reduced capacity to produce cytokines in response *in vitro* activation. Within the FRT, CD4+ T cells can produce IFNγ, IL-17A and IL-22 after activation. In the present study, we found that IFNγ production by CD4+ T cells from EM, CX, and ECX significantly declined as women age. Furthermore, IL-17A and IL-22 production by CD4+ T cells from ECX also significantly decreased with advancing age. Others have shown that IFNγ plays critical roles in tissue homeostasis, immune and inflammatory responses, offering protection against diseases by acting directly on target cells or through host immune system activation (59). Similarly, IL-17A functions as a key proinflammatory cytokine essential for defensing against bacterial and fungal infections, while IL-22 plays diverse roles in inflammation, mucous production, pathogen defense, wound healing, and tissue regeneration (60, 61). The decline in IFNγ, IL-17A and IL-22 production by CD4+ T cells throughout the FRT as women age suggests that CD4+ T cells ability to detect and eliminate pathogens is compromised. Although proinflammatory responses are crucial for combating infections, their dysregulation can lead to tissue damage (62). Our findings suggest that aging impacts CD4+ T cells’ ability within the FRT to provide essential immune protection, thereby influencing reproductive health and susceptibility to infections in aging women. Further investigation is warranted to analyze additional cytokines, including those involved in both proinflammatory and regulatory functions produced by CD4+ T cells in the FRT. It is also crucial to examine how age influences the secretion profiles of these cytokines by CD4+ T cells. Understanding these dynamics will provide deeper insights into the role of age in cytokine production and immune regulation.

Our findings demonstrate a previously unrecognized compartmentalization of FRT CD4+ T cell frequency and function with aging in the years following menopause. Aging following menopause leads to significant changes in FRT CD4+ T cell numbers, frequency, and phenotype. Our findings of enhanced CD69 expression with reduced capacity to produce cytokines by CD4+ T cells as women age following menopause suggests that FRT tissue resident memory CD4+ T cells contribute to the increased incidence of infections in the urogenital tract of post-menopausal women. Understanding the underlying factors and mechanisms involved in regulating immune protection by CD4+ T cells from FRT will provide a foundation of information essential for developing therapeutic tools to protect women against gynecological cancers and sexually transmitted infections as women age in the years following menopause.

## Data Availability

The original contributions presented in the study are included in the article/**Supplementary Material**. Further inquiries can be directed to the corresponding author.

## Supplementary material

Supplementary Figure 1 Cell viability. The percentage of viable cells, as determined by flow cytometry using dead cells staining, from a mixed cell suspension obtained after processing FRT tissue and removing dead cells. Two representative examples are shown.

Supplementary Figure 2 Menopause differentially regulates CD4+ T cell numbers and frequency in the FRT. **(A)** Comparison of pre- (black circle; EM: n=40, CX: n=17, ECX: n=15) vs. post-menopausal (white circle; EM: n=35, CX: n=16, ECX: n=15) women number of CD4+ T cells recovered per gram of tissue after magnetic bead isolation. **(B)** Comparison of pre- (black circle; EM: n=18, CX: n=19, ECX: n=19) and post-menopausal (white circle; EM: n=25, CX: n=18, ECX: n=20) women percentage of CD4+ T cells within CD3+ T cells. Each dot represents a single patient. Mean ± SEM are shown. *P<0.05, **P<0.01; Mann–Whitney U-test.

Supplementary Table 1 Surface monoclonal antibodies used for phenotyping.

Supplementary Table 2 Intracellular monoclonal antibodies used for phenotyping.

## References

[1] Bureau USC. United states census bureau, an aging world: 2015 United States census bureau (2016). Available online at: https://www.census.gov/library/publications/2016/demo/P95-16-1.html (accessed March 28, 2016).

[2] CDC. CDC, in surveillance supplemental report 2016 (2016). Available online at: https://www.cdc.gov/hiv/pdf/library/reports/surveillance/cdc-hiv-surveillance-report-2016-vol-28.pdf (accessed November 2017).

[3] CDC. Sexually transmitted diseases surveillance (2016). Available online at: https://www.cdc.gov/std/stats16/default.htm (accessed July 20, 2016).

[4] Wira CR Patel MV Ghosh M Mukura L Fahey JV. Innate immunity in the human female reproductive tract: endocrine regulation of endogenous antimicrobial protection against HIV and other sexually transmitted infections. Am J Reprod Immunol. (2011) 65:196–211. doi: 10.1111/j.1600-0897.2011.00970.x 21294805 PMC3837338

[5] Reis MaChado J da Silva MV Cavellani CL dos Reis MA Monteiro ML Teixeira Vde P. Mucosal immunity in the female genital tract, HIV/AIDS. BioMed Res Int. (2014) 2014:350195. doi: 10.1155/2014/350195 25313360 PMC4181941

[6] Wira CR Fahey JV Ghosh M Patel MV Hickey DK Ochiel DO. Sex hormone regulation of innate immunity in the female reproductive tract: the role of epithelial cells in balancing reproductive potential with protection against sexually transmitted pathogens. Am J Reprod Immunol. (2010) 63:544–65. doi: 10.1111/j.1600-0897.2010.00842.x PMC3837356 20367623

[7] Shifren JL Gass ML NAMS Recommendations for Clinical Care of Midlife Women Working Group. The North American Menopause Society recommendations for clinical care of midlife women. Menopause. (2014) 21:1038–62. doi: 10.1097/GME.0000000000000319 25225714

[8] Arias E Xu J Kochanek K. United States Life Tables, 2021. Natl Vital Stat Rep. (2023) 72(12):1–64. 38048433

[9] Walker ML Herndon JG. Menopause in nonhuman primates? Biol Reprod. (2008) 79(3):398–406. doi: 10.1095/biolreprod.108.068536 18495681 PMC2553520

[10] Rodriguez-Garcia M Patel MV Shen Z Wira CR. The impact of aging on innate and adaptive immunity in the human female genital tract. Aging Cell. (2021) 20:e13361. doi: 10.1111/acel.13361 33951269 PMC8135005

[11] Givan AL White HD Stern JE Colby E Gosselin EJ Guyre PM. Flow cytometric analysis of leukocytes in the human female reproductive tract: Comparison of Fallopian tube, uterus, cervix, and vagina. Am J Reprod Immunol. (1997) 38:350–9. doi: 10.1111/j.1600-0897.1997.tb00311.x 9352027

[12] Rodriguez-Garcia M Barr FD Crist SG Fahey JV Wira CR. Phenotype and susceptibility to HIV infection of CD4+ Th17 cells in the human female reproductive tract. Mucosal Immunol. (2014) 7:1375–85. doi: 10.1038/mi.2014.26 PMC4205172 24759207

[13] Rodriguez-Garcia M Fortier JM Barr FD Wira CR. Aging impacts CD103+ CD8+ T cell presence and induction by dendritic cells in the genital tract. Aging Cell. (2018) 17(3):e12733. doi: 10.1111/acel.12733 29455474 PMC5946085

[14] Meditz AL Moreau KL MaWhinney S Gozansky WS Melander K Kohrt WM. CCR5 expression is elevated on endocervical CD4+ T cells in healthy postmenopausal women. J Acquir Immune Defic Syndr. (2012) 59:221–8. doi: 10.1097/QAI.0b013e31823fd215 PMC3288615 22083068

[15] Saba E Grivel JC Vanpouille C Brichacek B Fitzgerald W Margolis L. HIV-1 sexual transmission: early events of HIV-1 infection of human cervico-vaginal tissue in an optimized ex vivo model. Mucosal Immunol. (2010) 3:280–90. doi: 10.1038/mi.2010.2 PMC3173980 20147895

[16] Masopust D Soerens AG. Tissue-resident T cells and other resident leukocytes. Annu Rev Immunol. (2019) 37:521–46. doi: 10.1146/annurev-immunol-042617-053214 PMC7175802 30726153

[17] Woodward Davis AS Vick SC Pattacini L Voillet V Hughes SM Lentz GM. The human memory T cell compartment changes across tissues of the female reproductive tract. Mucosal Immunol. (2021) 14:862–72. doi: 10.1038/s41385-021-00406-6 PMC8225572 33953338

[18] Rodriguez-Garcia M Shen Z Fortier JM Wira CR. Differential cytotoxic function of resident and non-resident CD8+ T cells in the human female reproductive tract before and after menopause. Front Immunol. (2020) 11:1096. doi: 10.3389/fimmu.2020.01096 32582183 PMC7287154

[19] Parthasarathy S Shen Z Carrillo-Salinas FJ Iyer V Vogell A Illanes D. Aging modifies endometrial dendritic cell function and unconventional double negative T cells in the human genital mucosa. Immun Ageing. (2023) 20:34. doi: 10.1186/s12979-023-00360-w 37452337 PMC10347869

[20] Shen Z Patel MV Rodriguez-Garcia M Wira CR. Aging beyond menopause selectively decreases CD8+ T cell numbers but enhances cytotoxic activity in the human endometrium. Immun Ageing. (2022) 19:55. doi: 10.1186/s12979-022-00312-w 36371240 PMC9652910

[21] Thome JJ Yudanin N Ohmura Y Kubota M Grinshpun B Sathaliyawala T. Spatial map of human T cell compartmentalization and maintenance over decades of life. Cell. (2014) 159:814–28. doi: 10.1016/j.cell.2014.10.026 PMC4243051 25417158

[22] Shen Z Rodriguez-Garcia M Patel MV Wira CR. Direct and Indirect endocrine-mediated suppression of human endometrial CD8+T cell cytotoxicity. Sci Rep. (2021) 11:1773. doi: 10.1038/s41598-021-81380-8 33469053 PMC7815780

[23] Rodriguez-Garcia M Fortier JM Barr FD Wira CR. Isolation of dendritic cells from the human female reproductive tract for phenotypical and functional studies. J Vis Exp. (2018) (133):57100. doi: 10.3791/57100 29608161 PMC5931758

[24] Rodriguez-Garcia M Shen Z Barr FD Boesch AW Ackerman ME Kappes JC. Dendritic cells from the human female reproductive tract rapidly capture and respond to HIV. Mucosal Immunol. (2017) 10:531–44. doi: 10.1038/mi.2016.72 PMC5332537 27579858

[25] Barr FD Ochsenbauer C Wira CR Rodriguez-Garcia M. Neutrophil extracellular traps prevent HIV infection in the female genital tract. Mucosal Immunol. (2018) 11:1420–8. doi: 10.1038/s41385-018-0045-0 PMC6162173 29875403

[26] Kumar BV Connors TJ Farber DL. Human T cell development, localization, and function throughout life. Immunity. (2018) 48:202–13. doi: 10.1016/j.immuni.2018.01.007 PMC5826622 29466753

[27] Kumar BV Ma W Miron M Granot T Guyer RS Carpenter DJ. Human tissue-resident memory T cells are defined by core transcriptional and functional signatures in lymphoid and mucosal sites. Cell Rep. (2017) 20:2921–34. doi: 10.1016/j.celrep.2017.08.078 PMC5646692 28930685

[28] Traxinger BR Richert-Spuhler LE Lund JM. Mucosal tissue regulatory T cells are integral in balancing immunity and tolerance at portals of antigen entry. Mucosal Immunol. (2022) 15:398–407. doi: 10.1038/s41385-021-00471-x 34845322 PMC8628059

[29] Jagger A Shimojima Y Goronzy JJ Weyand CM. Regulatory T cells and the immune aging process: a mini-review. Gerontology. (2014) 60:130–7. doi: 10.1159/000355303 PMC4878402 24296590

[30] Raynor J Lages CS Shehata H Hildeman DA Chougnet CA. Homeostasis and function of regulatory T cells in aging. Curr Opin Immunol. (2012) 24:482–7. doi: 10.1016/j.coi.2012.04.005 PMC3419320 22560294

[31] Agius E Lacy KE Vukmanovic-Stejic M Jagger AL Papageorgiou AP Hall S. Decreased TNF-alpha synthesis by macrophages restricts cutaneous immunosurveillance by memory CD4+ T cells during aging. J Exp Med. (2009) 206:1929–40. doi: 10.1084/jem.20090896 PMC2737169 19667063

[32] Hartigan-O’Connor DJ Poon C Sinclair E McCune JM. Human CD4+ regulatory T cells express lower levels of the IL-7 receptor alpha chain (CD127), allowing consistent identification and sorting of live cells. J Immunol Methods. (2007) 319:41–52. doi: 10.1016/j.jim.2006.10.008 17173927

[33] Kim GR Choi JM. Current understanding of cytotoxic T lymphocyte antigen-4 (CTLA-4) signaling in T-cell biology and disease therapy. Mol Cells. (2022) 45:513–21. doi: 10.14348/molcells.2022.2056 PMC9385567 35950451

[34] Walker LSK. EFIS Lecture: Understanding the CTLA-4 checkpoint in the maintenance of immune homeostasis. Immunol Lett. (2017) 184:43–50. doi: 10.1016/j.imlet.2017.02.007 28216262

[35] Acosta-Rodriguez EV Rivino L Geginat J Jarrossay D Gattorno M Lanzavecchia A. Surface phenotype and antigenic specificity of human interleukin 17-producing T helper memory cells. Nat Immunol. (2007) 8:639–46. doi: 10.1038/ni1467 17486092

[36] Zhu X Zhu J. CD4 T helper cell subsets and related human immunological disorders. Int J Mol Sci. (2020) 21(21):8011. doi: 10.3390/ijms21218011 33126494 PMC7663252

[37] Gray JI Westerhof LM MacLeod MKL. The roles of resident, central and effector memory CD4 T-cells in protective immunity following infection or vaccination. Immunology. (2018) 154:574–81. doi: 10.1111/imm.12929 PMC6050220 29570776

[38] Meter RA Wira CR Fahey JV. Secretion of monocyte chemotactic protein-1 by human uterine epithelium directs monocyte migration in culture. Fertility Sterility. (2005) 84:191–201. doi: 10.1016/j.fertnstert.2005.01.104 16009177

[39] Shen L Fahey JV Hussey SB Asin SN Wira CR Fanger MW. Synergy between IL-8 and GM–CSF in reproductive tract epithelial cell secretions promotes enhanced neutrophil chemotaxis. Cell Immunol. (2004) 230:23–32. doi: 10.1016/j.cellimm.2004.08.004 15541716

[40] Fahey JV Schaefer TM Channon JY Wira CR. Secretion of cytokines and chemokines by polarized human epithelial cells from the female reproductive tract. Hum Reproduction. (2005) 20:1439–46. doi: 10.1093/humrep/deh806 15734755

[41] Wira CR Rodriguez-Garcia M Shen Z Patel M Fahey JV. The role of sex hormones and the tissue environment in immune protection against HIV in the female reproductive tract. Am J Reprod Immunol. (2014) 72:171–81. doi: 10.1111/aji.12235 PMC4106999 24661500

[42] Lund JM Hladik F Prlic M. Advances and challenges in studying the tissue-resident T cell compartment in the human female reproductive tract. Immunol Rev. (2023) 316:52–62. doi: 10.1111/imr.13212 37140024 PMC10524394

[43] Turner DL Farber DL. Mucosal resident memory CD4 T cells in protection and immunopathology. Front Immunol. (2014) 5:331. doi: 10.3389/fimmu.2014.00331 25071787 PMC4094908

[44] Mueller SN Mackay LK. Tissue-resident memory T cells: local specialists in immune defence. Nat Rev Immunol. (2016) 16:79–89. doi: 10.1038/nri.2015.3 26688350

[45] Sathaliyawala T Kubota M Yudanin N Turner D Camp P Thome JJ. Distribution and compartmentalization of human circulating and tissue-resident memory T cell subsets. Immunity. (2013) 38:187–97. doi: 10.1016/j.immuni.2012.09.020 PMC3557604 23260195

[46] Mackay LK Braun A Macleod BL Collins N Tebartz C Bedoui S. Cutting edge: CD69 interference with sphingosine-1-phosphate receptor function regulates peripheral T cell retention. J Immunol. (2015) 194:2059–63. doi: 10.4049/jimmunol.1402256 25624457

[47] Mackay LK Stock AT Ma JZ Jones CM Kent SJ Mueller SN. Long-lived epithelial immunity by tissue-resident memory T (TRM) cells in the absence of persisting local antigen presentation. Proc Natl Acad Sci U S A. (2012) 109:7037–42. doi: 10.1073/pnas.1202288109 PMC3344960 22509047

[48] Steinert EM Schenkel JM Fraser KA Beura LK Manlove LS Igyarto BZ. Quantifying memory CD8 T cells reveals regionalization of immunosurveillance. Cell. (2015) 161:737–49. doi: 10.1016/j.cell.2015.03.031 PMC4426972 25957682

[49] Szabo PA Miron M Farber DL. Location, location, location: Tissue resident memory T cells in mice and humans. Sci Immunol. (2019) 4(34):eaas9673. doi: 10.1126/sciimmunol.aas9673 30952804 PMC6778482

[50] Traxinger B Vick SC Woodward-Davis A Voillet V Erickson JR Czartoski J. Mucosal viral infection induces a regulatory T cell activation phenotype distinct from tissue residency in mouse and human tissues. Mucosal Immunol. (2022) 15:1012–27. doi: 10.1038/s41385-022-00542-7 PMC9391309 35821289

[51] Thome JJ Bickham KL Ohmura Y Kubota M Matsuoka N Gordon C. Early-life compartmentalization of human T cell differentiation and regulatory function in mucosal and lymphoid tissues. Nat Med. (2016) 22:72–7. doi: 10.1038/nm.4008 PMC4703455 26657141

[52] Abu-Raya B Michalski C Sadarangani M Lavoie PM. Maternal immunological adaptation during normal pregnancy. Front Immunol. (2020) 11:575197. doi: 10.3389/fimmu.2020.575197 33133091 PMC7579415

[53] Patel MV Shen Z Wira CR. Do endometrial immune changes with age prior to menopause compromise fertility in women? Explor Immunol. (2022) 2:677–92. doi: 10.37349/ei.2022.00076 PMC11809571 39931230

[54] Huang N Chi H Qiao J. Role of regulatory T cells in regulating fetal-maternal immune tolerance in healthy pregnancies and reproductive diseases. Front Immunol. (2020) 11:1023. doi: 10.3389/fimmu.2020.01023 32676072 PMC7333773

[55] Wira CR Fahey JV. The innate immune system: gatekeeper to the female reproductive tract. Immunology. (2004) 111:13–5. doi: 10.1111/j.1365-2567.2004.01796.x PMC1782397 14678193

[56] Ssemaganda A Cholette F Perner M Kambaran C Adhiambo W Wambugu PM. Endocervical regulatory T cells are associated with decreased genital inflammation and lower HIV target cell abundance. Front Immunol. (2021) 12:726472. doi: 10.3389/fimmu.2021.726472 34630402 PMC8495419

[57] Saito S Tsukaguchi N Hasegawa T Michimata T Tsuda H Narita N. Distribution of Th1, Th2, and Th0 and the Th1/Th2 cell ratios in human peripheral and endometrial T cells. Am J Reprod Immunol. (1999) 42:240–5. doi: 10.1111/j.1600-0897.1999.tb00097.x 10580606

[58] Kamali AN Noorbakhsh SM Hamedifar H Jadidi-Niaragh F Yazdani R Bautista JM. A role for Th1-like Th17 cells in the pathogenesis of inflammatory and autoimmune disorders. Mol Immunol. (2019) 105:107–15. doi: 10.1016/j.molimm.2018.11.015 30502718

[59] Jorgovanovic D Song M Wang L Zhang Y. Roles of IFN-gamma in tumor progression and regression: a review. biomark Res. (2020) 8:49. doi: 10.1186/s40364-020-00228-x 33005420 PMC7526126

[60] Curtis MM Way SS. Interleukin-17 in host defence against bacterial, mycobacterial and fungal pathogens. Immunology. (2009) 126:177–85. doi: 10.1111/j.1365-2567.2008.03017.x PMC2632692 19125888

[61] Arshad T Mansur F Palek R Manzoor S Liska V. A double edged sword role of interleukin-22 in wound healing and tissue regeneration. Front Immunol. (2020) 11:2148. doi: 10.3389/fimmu.2020.02148 33042126 PMC7527413

[62] Chen L Deng H Cui H Fang J Zuo Z Deng J. Inflammatory responses and inflammation-associated diseases in organs. Oncotarget. (2018) 9:7204–18. doi: 10.18632/oncotarget.v9i6 PMC5805548 29467962

