# Supplementary figures and images for: Impact of aging on the frequency, phenotype, and function of CD4+ T cells in the human female reproductive tract
Source: Front Immunol. 2024 Sep 12;15:1465124. doi: 10.3389/fimmu.2024.1465124 (PMC11424415; doi:10.3389/fimmu.2024.1465124)

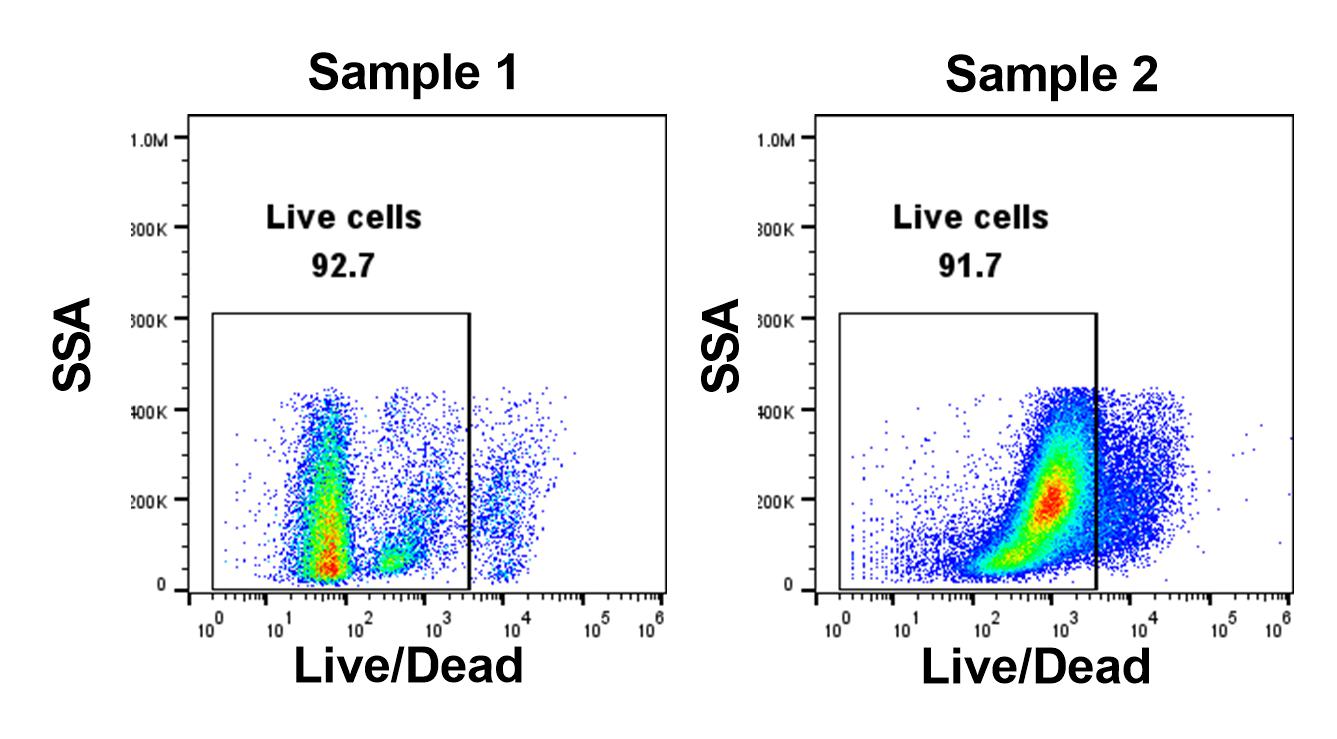

Supplement: Supplementary Figure 1 — Cell viability. The percentage of viable cells, as determined by flow cytometry using dead cells staining, from a mixed cell suspension obtained after processing FRT tissue and removing dead cells. Two representative examples are shown. [file Image1.jpeg]

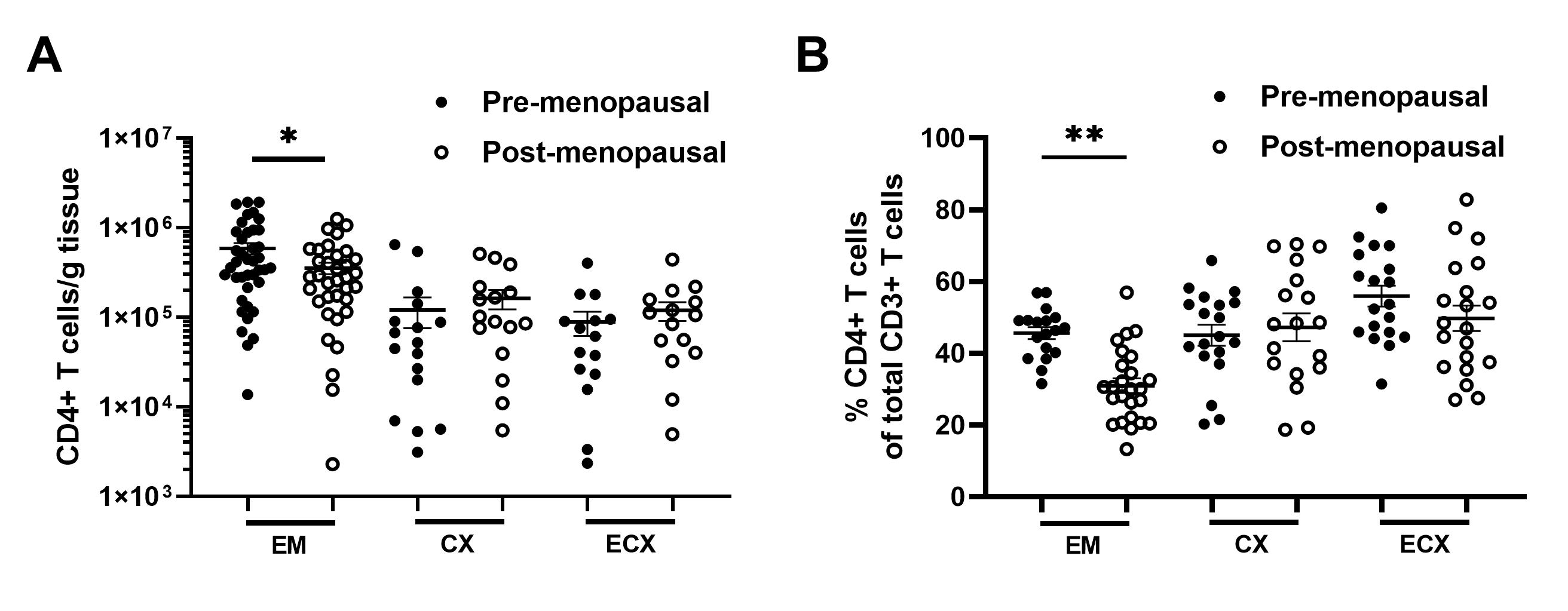

Supplement: Supplementary Figure 2 — Menopause differentially regulates CD4+ T cell numbers and frequency in the FRT. (A) Comparison of pre- (black circle; EM: n=40, CX: n=17, ECX: n=15) vs. post-menopausal (white circle; EM: n=35, CX: n=16, ECX: n=15) women number of CD4+ T cells recovered per gram of tissue after magnetic bead isolation. (B) Comparison of pre- (black circle; EM: n=18, CX: n=19, ECX: n=19) and post-menopausal (white circle; EM: n=25, CX: n=18, ECX: n=20) women percentage of CD4+ T cells within CD3+ T cells. Each dot represents a single patient. Mean ± SEM are shown. *P<0.05, **P<0.01; Mann–Whitney U-test. [file Image2.jpeg]
