# Supplementary material for: Impact of aging on the frequency, phenotype, and function of CD4+ T cells in the human female reproductive tract
Source: Front Immunol. 2024 Sep 12;15:1465124. doi: 10.3389/fimmu.2024.1465124 (PMC11424415; doi:10.3389/fimmu.2024.1465124)
Supplement: Supplementary Table 1 — Surface monoclonal antibodies used for phenotyping. [file Table1.docx]

**Supplementary Table 1. Surface monoclonal antibodies used for phenotyping.**

| **Anti Human** | **clone** | **fluorochrome** | **Catalog** | **manufacturer** |
| --- | --- | --- | --- | --- |
| **CD3** | UCHT1 | APC/Cy7 | 300426 | BioLegend |
| **CD3** | SK7 | e450 | 48-0036-42 | eBioscience^TM^ |
| **CD4** | OKT4 | FITC | 11-0048-42 | eBioscience^TM^ |
| **CD4** | OKT4 | PE | 317410 | BioLegend |
| **CD8** | SK1 | e450 | 75-0087-T100 | Tonbo |
| **CD8** | SK1 | FITC | 344703 | BioLegend |
| **CD25** | BC96 | APC/Cy7 | 302613 | BioLegend |
| **CD45** | 2D1 | AF700 | 368514 | BioLegend |
| **CD69** | FN50 | BV510 | 310936 | BioLegend |
| **CD103** | B-Ly7 | PE/Cy7 | 25-1038-42 | eBioscience^TM^ |
| **CD127** | A019D5 | APC | 351316 | BioLegend |
| **CXCR3 (CD183)** | G025H7 | PE/Cyanine5 | 353755 | BioLegend |
| **CCR4 (CD194)** | L291H4 | PE/Cyanine7 | 359410 | BioLegend |
| **CCR5 (CD195)** | J418F1 | PerCP/Cyanine5.5 | 359111 | BioLegend |
| **CCR5 (CD195)** | HEK/1/85a | FITC | 313705 | BioLegend |
| **CCR6 (CD196)** | G034E3 | PE/Dazzle^TM^ 594 | 353429 | BioLegend |
