# Supplementary material for: Impact of aging on the frequency, phenotype, and function of CD4+ T cells in the human female reproductive tract
Source: Front Immunol. 2024 Sep 12;15:1465124. doi: 10.3389/fimmu.2024.1465124 (PMC11424415; doi:10.3389/fimmu.2024.1465124)
Supplement: Supplementary Table 2 — Intracellular monoclonal antibodies used for phenotyping. [file Table2.docx]

**Supplementary Table 2. Intracellular monoclonal antibodies used for phenotyping.**

| **Anti Human** | **clone** | **fluorochrome** | **Catalog** | **manufacturer** |
| --- | --- | --- | --- | --- |
| **CTLA-4 (CD152)** | BNI3 | BV421 | 369606 | BioLegend |
| **IL-4** | MP4-25D2 | APC | 500811 | BioLegend |
| **IL-17A** | BL168 | PE/Dazzle^TM^ 594 | 512335 | BioLegend |
| **IL-22** | HEK/1/85a | PE/Cyanine7 | 366707 | BioLegend |
| **IFNγ** | G034E3 | PerCP/Cyanine5.5 | 353429 | BioLegend |
